# A multi-segment underwater acoustic array telemetry dataset with depth, heading, pitch, and roll time series

**DOI:** 10.1016/j.dib.2026.113143

**Published:** 2026-08-06

**Authors:** Fujin Huang, Lei Zhao

**Affiliations:** a Dalian Scientific Test and Control Technology Institute, Dalian, China; b Dalian Neusoft University of Information, Dalian, China

**Keywords:** Underwater sensor array, Depth time series, Orientation telemetry, Array geometry, Marine instrumentation, Attitude data

## Abstract

This data article presents time-series telemetry datasets collected from underwater acoustic sensor arrays during field deployments. The data comprise synchronized measurements of depth (m), heading (°), pitch (°), and roll (°), recorded at a nominal sampling rate of approximately 1 Hz and provided in comma-separated value (CSV) format with time-of-day timestamps and deployment location identifiers. The release includes two related CSV files covering different array configurations and water-depth conditions, enabling comparative analysis of array geometry dynamics, sensor orientation stability, and depth variation patterns. An accompanying browser-based 3D visualization tool supports rapid data validation and interactive exploration without specialized software. These datasets are intended for researchers in underwater instrumentation, ocean engineering, and geophysical array deployment who require reference telemetry for array behavior analysis, algorithm development, or educational purposes. The data and visualization tool are deposited in a public repository under an open license, with documented file structure and column definitions.

Specifications Table

**Table:** 

Subject	Earth & Environmental Sciences
Specific subject area	Underwater acoustic sensor array telemetry; time-series analysis of array geometry and orientation
Type of data	Raw: CSV time-series file (.csv); Table: Structured sensor readings grouped by timestamp
Data collection	Sensor telemetry data were collected from underwater acoustic sensor arrays during a continuous surface-logging campaign (∼27 h, from 08:08 to 11:04 the following day) and are released as two CSV files representing different deployment configurations. Synchronized depth, heading, pitch, and roll measurements were recorded at approximately 1 Hz from sensors on multiple submerged segments (Low-frequency Array-L1–L3 and Mid-frequency Array-L4 in the primary file; Mid-frequency Array-L4 and L6 in the secondary file). Data were logged at the surface receiving system and exported as CSV records with time-of-day timestamps and deployment location identifiers.
Data source location	Institution: No. 760 Research Institute, China State Shipbuilding Corporation Limited (CSSC), Dalian, China
Data accessibility	Repository name: figshare Data identification number: 10.6084/m9.figshare.32,776,920 Direct URL to data: 10.6084/m9.figshare.32,776,920
Related research article	none

## 1 Value of the Data

This dataset is particularly valuable for researchers developing and benchmarking sensor fusion, Kalman filtering, and array shape estimation algorithms, machine learning-based predictive modeling, anomaly detection, and time-series forecasting. The following five points summarize the specific contributions of this release.

**Relevance to ocean engineering and navigation applications:** In practical ocean engineering and navigation scenarios, the synchronized depth and attitude records can support underwater vehicle localization by providing reference trajectories for dead-reckoning correction, aid in the deployment and recovery of tethered sensor arrays through geometric stability monitoring, and enable predictive maintenance by detecting anomalous sensor behavior indicative of incipient instrument failure.

**Scarcity of public array-geometry telemetry:** Public underwater telemetry datasets containing synchronized depth–heading–pitch–roll measurements from multiple array segments are extremely scarce [1]. Existing public marine datasets typically focus on acoustic recordings, oceanographic parameters, or vehicle trajectories, whereas telemetry datasets describing the geometric behavior of deployed acoustic arrays are rarely released. The present release addresses this gap by providing multi-segment, multi-configuration attitude and depth records from a continuous field campaign, enabling demonstrated comparative analysis of array geometry dynamics rather than hypothetical reuse scenarios alone.

**Extended-duration, multi-configuration array geometry reference:** These datasets provide approximately 27 h of continuous, synchronized depth and orientation (heading, pitch, roll) measurements from underwater acoustic sensor arrays released in two related CSV files. The primary file covers a multi-segment deployment with low-frequency and mid-frequency sub-arrays (Low-frequency Array-L1, L2, L3; Mid-frequency Array-L4), while the secondary file covers a complementary configuration on two mid-frequency segments (Mid-frequency Array-L4 and L6). Together, they span shallow to deeper operating depths and offer an openly available reference for studying dynamic array geometry, inter-segment variability, and temporal stability under field deployment conditions [2].

**Raw telemetry for algorithm development and validation:** The data are presented in raw, unprocessed form as recorded by the surface telemetry system. No filtering, smoothing, or interpolation has been applied to the depth, heading, pitch, or roll values. This makes the datasets suitable for developing or validating methods such as outlier detection, sensor fusion, Kalman filtering [3], and orientation estimation [4,5]. Four attitude-related variables per sensor support testing of attitude determination and array shape estimation workflows.

**Comparative data across deployment configurations and segment types:** Because the release includes two array configurations logged within the same continuous acquisition campaign, users can compare depth maintenance, orientation behavior, and segment-level response across different sub-array types (low-frequency vs. mid-frequency) and across shallow vs. deeper deployment conditions. This supports configuration-dependent benchmarking without requiring users to assemble data from separate sources.

**Accessible format with integrated visualization utility:** The datasets are provided in standard comma-separated value (CSV) format with a documented header structure, ensuring compatibility with spreadsheet software and common analysis environments (Python, MATLAB, R). A supplementary browser-based 3D visualization tool enables rapid visual inspection, data quality assessment, and intuitive exploration of array geometry dynamics, lowering the barrier to reuse for researchers and students without specialized software.

## 2 Background

Underwater acoustic sensor arrays are widely used in marine geophysical exploration, oceanographic monitoring, and distributed sensing applications, where accurate knowledge of sensor position and orientation is essential for beamforming, source localization, and array signal processing [6,7]. In addition to acoustic measurements, the dynamic geometry and attitude of deployed arrays can significantly influence signal quality and downstream inversion accuracy.

Recent studies in underwater sensing systems have increasingly emphasized the importance of attitude-aware modelling and sensor fusion for improving array stability estimation and navigation robustness. For example, IMU-assisted underwater navigation and orientation estimation methods have been widely explored in autonomous underwater vehicles and tethered sensor systems, highlighting the role of high-frequency attitude telemetry in correcting drift and improving spatial consistency [[8], [9], [10], [11]]. More recent work has also focused on integrating multi-sensor fusion frameworks and time-synchronized telemetry streams to enhance robustness in complex marine environments [1,12].

Despite these advances, publicly available datasets that provide synchronized multi-segment underwater array telemetry remain limited. Existing open marine datasets are typically focused on acoustic recordings, vehicle trajectories, or environmental parameters such as temperature and salinity, while datasets capturing long-duration, multi-sensor attitude (depth, heading, pitch, roll) dynamics of fixed or semi-fixed underwater arrays are rarely released [1,13,14]. In particular, there is a lack of benchmark datasets that allow direct comparison of array geometry behavior across different deployment configurations under continuous field conditions.

To address this gap, we present a time-series telemetry dataset collected from an underwater acoustic sensor array during a continuous field deployment of approximately 27 h [15]. The dataset includes synchronized measurements of depth, heading, pitch, and roll at ∼1 Hz sampling frequency across multiple array segments and two deployment configurations. The release preserves raw, unprocessed telemetry streams, enabling reproducibility and flexible downstream analysis.

The dataset is particularly suitable for research on sensor fusion, attitude smoothing, outlier detection, Kalman filtering, and array geometry reconstruction, as well as for machine learning-driven predictive modeling and time-series forecasting of array behavior. In addition, the inclusion of co-located sensor pairs allows internal consistency validation of telemetry signals, providing an additional reference for quality assessment and algorithm benchmarking. A browser-based 3D visualization tool is also provided to facilitate intuitive exploration of array dynamics without requiring specialized software.

Overall, this dataset contributes a rare publicly available reference for multi-segment underwater array telemetry, enabling both methodological development and comparative benchmarking in underwater sensing and marine instrumentation research.

## 3 Data Description

The released dataset is publicly available through figshare and consists of two CSV files representing two deployment configurations of the underwater acoustic sensor array, as summarized in Table 1.

**Table 1:** File inventory for the dataset.

File Name	Format	Description
Dataset A (underwater_sensor_array_8.csv)	CSV	Time-series telemetry from an **8-sensor** multi-segment array configuration (**Low-frequency Array-L1, L2, L3; Mid-frequency Array-L4**; sensors Y23_T23–Y30_T30). Contains synchronized depth (m), heading (°), pitch (°), and roll (°) at ∼1 Hz (∼30,479 timestamps; ∼8.5 h). Deeper operating depths (∼13–51 m).
Dataset B (underwater_sensor_array_4.csv)	CSV	Time-series telemetry from a **4-sensor** mid-frequency array configuration (**Mid-frequency Array-L4** and **L6**; sensors Y29_T29–Y32_T32). Contains synchronized depth (m), heading (°), pitch (°), and roll (°) at ∼1 Hz (∼49,557 timestamps; ∼13.8 h). Shallow-water deployment (∼12–18 m sensor depths).
README.md	Markdown	Documentation file with column definitions, sensor assignment tables, usage notes, data quality summary, and citation information.

**Data files:** Both data files are formatted as comma-separated value (CSV) files with UTF- 8 encoding. The first row contains column headers. Each subsequent row represents a single sensor reading at a specific timestamp. The two files share an identical column structure and differ only in array configuration, sensor inventory, and deployment conditions (see Table 1). Table 2 provides the column definitions applicable to both files.

**Table 2:** Column definitions for the primary data file.

Column Name	Data Type	Description	Example Value
Reception Time	String (H:MM:SS or HH:MM:SS)	Time of data reception at the surface logging system, assigned upon demultiplexing of the telemetry stream. Due to the negligible transmission latency of the cable-based telemetry link, this timestamp is effectively equivalent to the measurement acquisition time. Time-of-day only; date not included	8:08:12
Deployment Location	String	Array segment identifier	Low-frequency Array-L1
Sensor Name	String	Unique sensor identifier	Y23_T23
Depth (m)	Float	Sensor depth relative to the water surface; negative values indicate below the surface	−36.0099
Heading (°)	Float	Compass heading; 0° = North, 90° = East	134.71
Pitch (°)	Float	Nose-up/nose-down orientation; positive = nose-up	24.6299
Roll (°)	Float	Port/starboard tilt; approximately −180° to +180°	−149.22

### 3.1 Sensor array configuration

The release includes two array configurations (Table 1). In Dataset A (underwater_sensor_array_8.csv), eight sensors are deployed at four equally spaced positions along the array. Two sensors are co-located at each position, yielding eight independent telemetry channels. In Dataset B (underwater_sensor_array_4.csv), four sensors are deployed at two positions, with two co-located sensors at each position. Table 3, Table 4 summarize sensor assignments by position (deployment location).

**Table 3:** Sensor assignment by array segment (Dataset A).

Segment	Array Type	Co-located Sensor A	Co-located Sensor B
L1	Low-frequency	Y23_T23	Y24_T24
L2	Low-frequency	Y25_T25	Y26_T26
L3	Low-frequency	Y27_T27	Y28_T28
L4	Mid-frequency	Y29_T29	Y30_T30

**CSV field:** Deployment Location = Low-frequency Array-L1 ... Mid-frequency Array-L4. The four positions are equally spaced along the array with an inter-position distance of approxi- mately 1.5 m; exact inter-position distances are not included in the dataset due to operational confidentiality constraints.

**Table 4:** Sensor assignment by array segment (Dataset B).

Segment	Array Type	Co-located Sensor A	Co-located Sensor B
L4	Mid-frequency	Y29_T29	Y30_T30
L6	Mid-frequency	Y31_T31	Y32_T32

## 4 Experimental Design, Materials and Methods

### 4.1 Array configuration and deployment

**Fig. 1:**
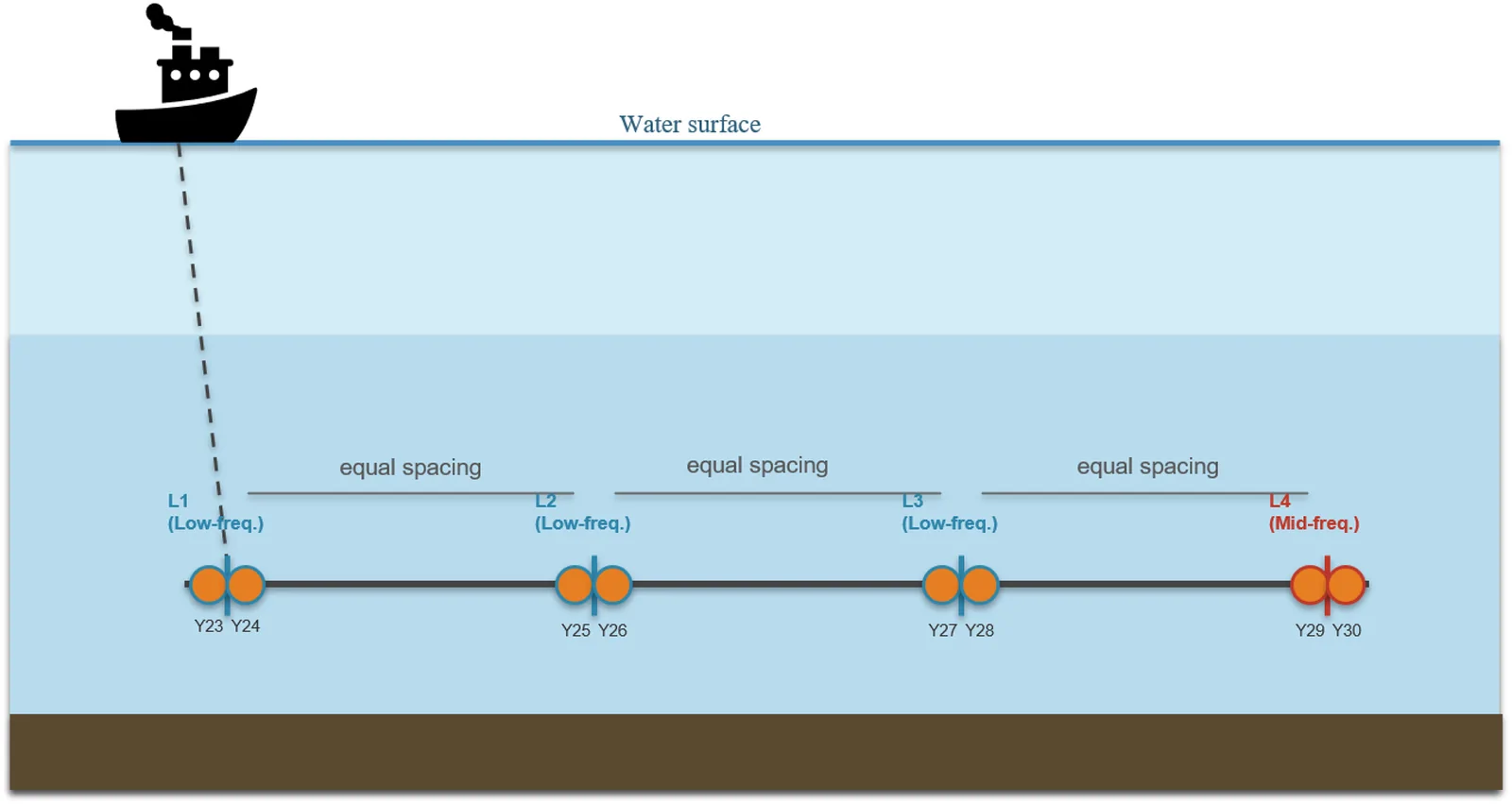
Schematic of the eight-sensor underwater acoustic array configuration. The array comprises four equally spaced positions (L1–L4); two sensors are co-located at each position (eight sensors total). Positions L1–L3 are low-frequency sub-arrays; L4 is a mid-frequency sub-array. Dashed line: telemetry link to the surface logging system.

This subsection describes the array configuration recorded in Dataset A (underwater_sensor_array_8.csv). The underwater acoustic sensor array comprised four equally spaced deployment positions along the submerged array (designated L1, L2, L3, and L4 in the telemetry records). Two sensor units were co-located at each position, yielding eight independent telemetry channels in total. Positions L1–L3 correspond to low-frequency sub-arrays; position L4 corresponds to a mid-frequency sub-array. The array was deployed under deep-water conditions during the eight-sensor logging phase. Sensor assignments by position are summarized in Table 3 and depicted schematically in Fig. 1.

### 4.2 Sensor instrumentation

Each sensor unit transmitted depth, heading, pitch, and roll through measurement components typical of marine telemetry systems used on submerged acoustic arrays. Depth was derived from integrated pressure sensing; heading from a triaxial magnetometer/compass; pitch and roll from a MEMS-based inertial measurement unit (IMU). Table 5 summarizes the measurement variables and associated sensor types.

**Table 5:** Summary of sensor measurement components and typical specifications.

Measurement	Sensor Type	Output Range	Output Rate
Depth	Piezoresistive pressure sensor	Negative values (m below surface)	1 Hz
Heading	Triaxial magnetometer	0° to 360°	1 Hz
Pitch	MEMS inertial measurement unit	−90° to +90°	1 Hz
Roll	MEMS inertial measurement unit	−180° to +180°	1 Hz

The sensor modules are commercial off-the-shelf marine-grade instrumentation with factory-calibrated performance. Specific manufacturer identities, model numbers, and individual unit calibration records are not disclosed to comply with institutional data-sharing policies. All values in the released CSV files are raw telemetry readings; no accuracy corrections, bias removal, or temperature compensation have been applied. This unprocessed format preserves the original measurement stream as acquired during the field campaign and supports independent algorithm development and sensor fusion benchmarking. Although factory calibration coefficients are not provided, the co-located sensor pairs at each deployment position offer an internal reference for assessing measurement consistency and performing empirical relative calibration, as demonstrated in the co-located sensor analysis (Table 12). For general reference, comparable commercial-grade MEMS pressure sensors typically achieve an accuracy of ±0.25% FS, and fiber optic IMUs commonly used in such applications offer bias stability on the order of 0.05°/h.

**Table 13:** Moving-average smoothing metrics for sensor Y29_T29 (Dataset A).

Metric	Value
Raw pitch SD (σ_raw)	19.68°
Smoothed pitch SD (σ_smooth)	19.64°
Residual SD (raw − smoothed)	1.13°
Peak |raw − smoothed|	11.35°
Mean pitch (before / after)	82.73° / 82.73°

### 4.3 Data acquisition system and telemetry

**Fig. 2:**
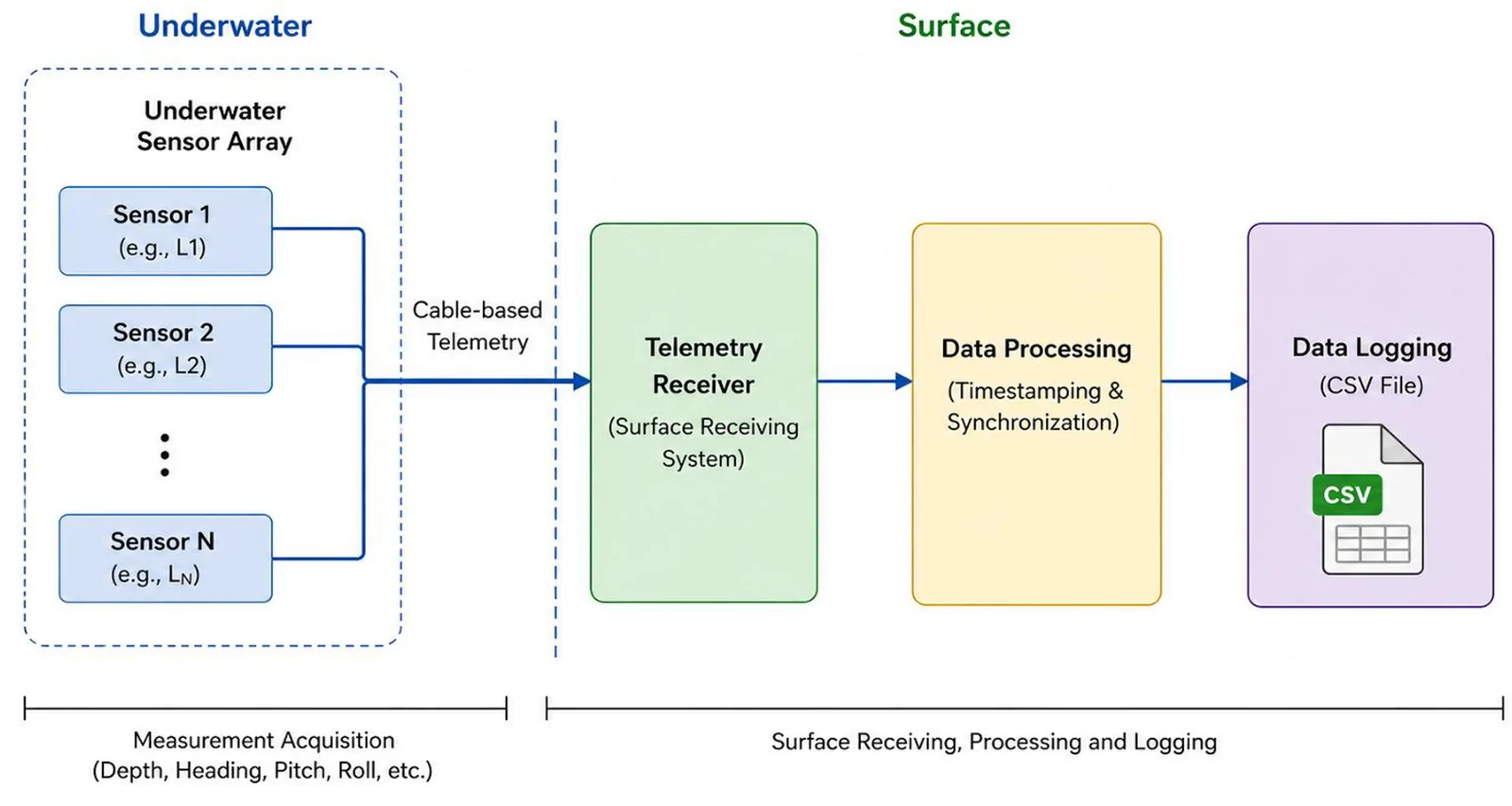
Schematic of the data acquisition system architecture. Measurements from the underwater sensor array are transmitted via cable-based telemetry to the surface receiving system, where they are timestamped, synchronized, and logged to CSV files.

The data acquisition system comprised three primary functional modules: the underwater sensor array, the cable-based telemetry transmission link, and the surface receiving and logging unit. Fig. 2 illustrates the data flow for the eight-sensor logging configuration (Dataset A (underwater_sensor_array_8.csv)). The same architecture was used during the four-sensor logging phase, with four active sensor channels exported to Dataset B (underwater_sensor_array_4.csv). Table 6 summarizes the processing stages from sensor measurement to CSV export.

**Table 6:** Data flow stages in the acquisition system architecture.

Stage	Component	Function	Output
1	Underwater sensors (×8)	Acquire depth, heading, pitch, roll	Sensor readings
2	Multiplexer (subsea)	Combine 8 sensor streams	Multiplexed data stream
3	Cable telemetry link	Transmit data to surface	Electrical/optical signal
4	Surface receiver	Demultiplex and timestamp	Individual sensor records
5	Logging software	Format and write to disk	CSV file (raw dataset)

**Sampling and telemetry parameters:** Measurements were acquired synchronously at a nominal interval of approximately 1 s (∼1 Hz). Sensor data were transmitted in real time to the surface receiving system through a cable-based, multiplexed telemetry link integrated with the deployment cable. Time-division multiplexing (TDM) was used to combine multiple sensor streams into a single transmission channel.

**Surface logging:** At the surface, a logging computer received the multiplexed stream. Receiver software demultiplexed the data into individual sensor channels, assigned a synchronized 1 s timestamp to each record using the system clock, and appended rows to UTF-8-encoded CSV files on local storage. The timestamp reflects the reception time at the surface system; transmission latency over the cable-based link is negligible (∼milliseconds), making the reception time effectively equivalent to the measurement acquisition time. Logging was continuous over the field campaign from 08:08 on Day 1 to 11:04 on Day 2 (∼27 h wall-clock period), producing two related output files (Table 1). Table 7 summarizes acquisition parameters; Table 8 summarizes coverage by file.

**Table 7:** Data acquisition parameters.

Parameter	Value
Sampling interval	∼1 s
Sampling frequency	∼1 Hz
Telemetry method	Cable-based multiplexed transmission
Multiplexing technique	Time-division multiplexing (TDM)
Timestamp resolution	1 s (time-of-day; date not stored in CSV)
Data format	Raw CSV (UTF-8), no post-processing
File encoding	UTF-8
Output files	Dataset A (underwater_sensor_array_8.csv), Dataset B (underwater_sensor_array_4.csv)

**Table 8:** Data coverage summary.

Parameter	Dataset A	Dataset B
Active sensors	8 (Y23_T23–Y30_T30)	4 (Y29_T29–Y32_T32)
Array positions	4 (L1–L4)	2 (L4, L6)
Sensors per position	2 (co-located)	2 (co-located)
Timestamps	30,479	49,557
Records (rows)	243,753	196,288
Sampled duration (∼)	∼8.5 h	∼13.8 h
Sensor depth range (m)	∼−13 to −51	∼−12 to −18
Nominal deployment depth	Deep-water deployment	Shallow-water deployment

**Note:** The ∼27 h wall-clock interval from 08:08 (Day 1) to 11:04 (Day 2) represents the total duration of the field campaign, which included equipment reconfiguration between the two logging phases. Dataset A (∼8.5 h) and Dataset B (∼13.8 h) correspond to the two active logging periods within this campaign; the difference between the total campaign duration and the sum of the two logging periods reflects setup, reconfiguration, and idle time between deployments.

### 4.4 Deployment protocol

Data were recorded during two active logging phases within a continuous field campaign spanning from 08:08 on Day 1 to 11:04 on Day 2 (∼27 h total campaign duration). The campaign included equipment reconfiguration between the two phases, resulting in two discrete CSV outputs rather than a single continuous file. Dataset A documents the eight-sensor configuration at four equally spaced positions (L1–L4) under deep-water deployment conditions. Dataset B documents a subsequent four-sensor configuration on two mid-frequency positions (L4 and L6) under shallow-water deployment conditions. Table 8 summarizes file-specific coverage.

### 4.5 Dataset overview and descriptive statistics

Table 9, Table 10 summarize descriptive statistics (mean, standard deviation, minimum, maximum) for all measurement variables in Dataset A and Dataset B, respectively. These statistics were computed from the released CSV files without filtering or smoothing.

**Table 9:** Descriptive statistics for Dataset A (underwater_sensor_array_8.csv).

Variable	Mean	SD	Min	Max
Depth	−37.83	8.94	−50.92	−12.04
Heading	158.52	64.98	0.10	359.99
Pitch	16.92	47.52	−59.70	90.00
Roll	−11.28	73.88	−179.96	179.96

**Table 10:** Descriptive statistics for Dataset B (underwater_sensor_array_4.csv).

Variable	Mean	SD	Min	Max
Depth	−14.11	1.24	−18.16	−12.55
Heading	197.79	94.12	2.30	340.90
Pitch	0.57	2.58	−110.90	9.78
Roll	−42.20	73.00	−115.90	122.35

**Note:** The Dataset B pitch minimum (−110.90°) exceeds the nominal ±90° sensor range in Table 5; this value is retained in the raw release (see Range validation). Such values may arise from momentary sensor anomalies, electromagnetic interference, or initialization artifacts in the MEMS inertial measurement unit; users should treat this as a raw outlier and apply appropriate filtering (e.g., threshold-based removal or median filtering) depending on their specific application (see also Range validation in the Data Quality Validation section).

### 4.6 Representative data visualizations

**Fig. 3:**
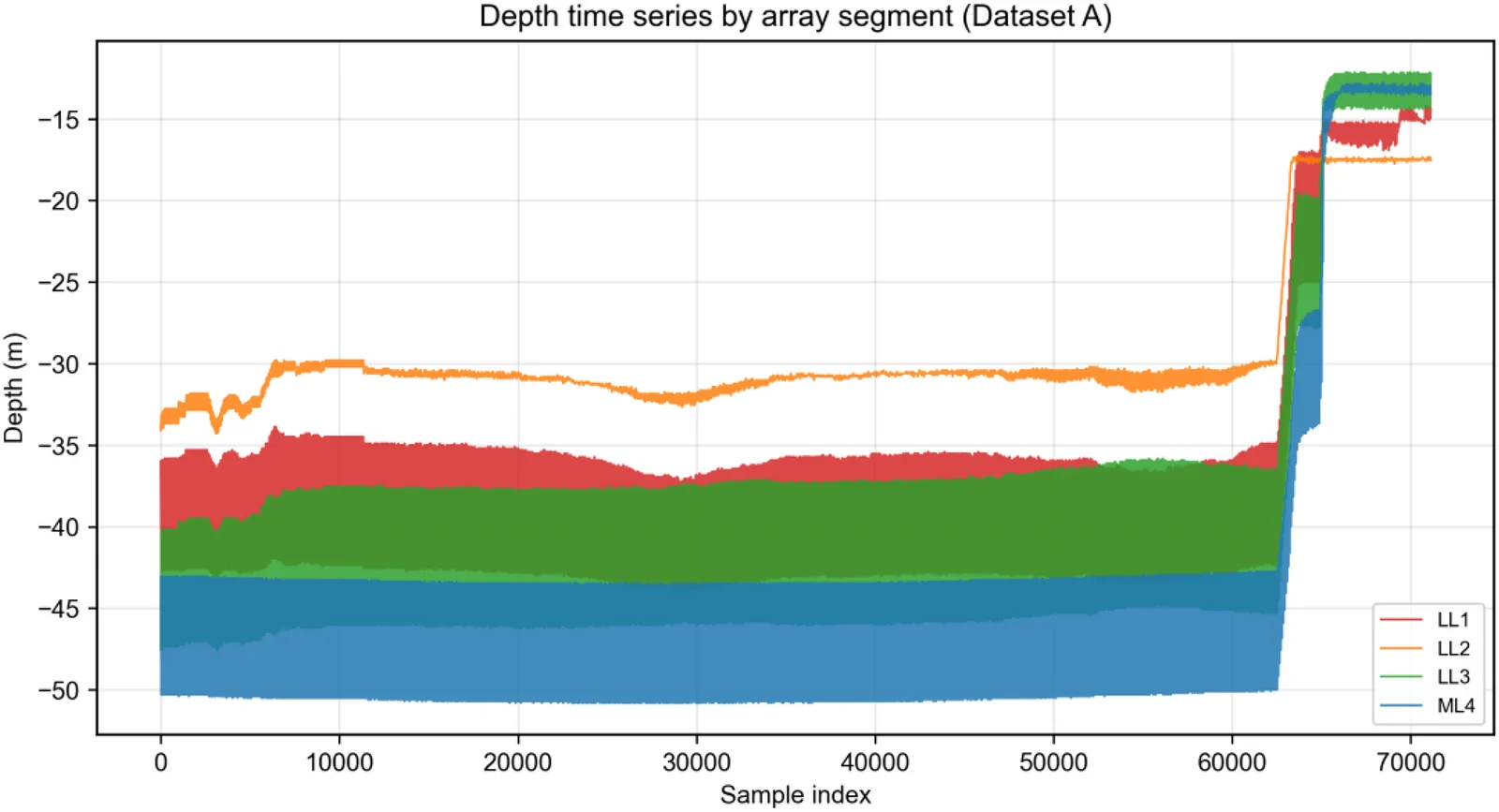
Depth time series by array segment (Dataset A). Co-located sensors at each position are plotted independently.

**Fig. 4:**
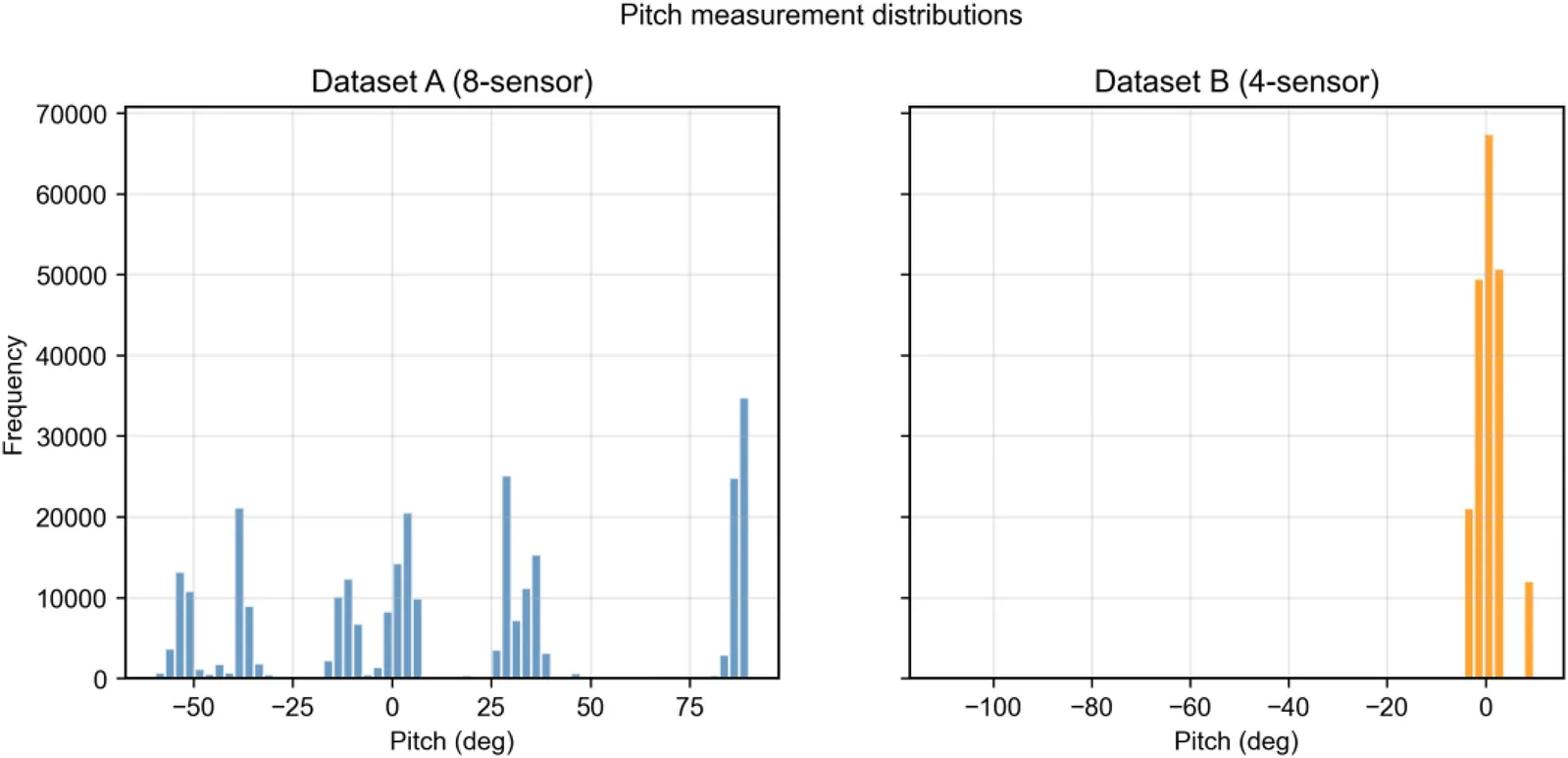
Pitch measurement distributions for Dataset A (left) and Dataset B (right).

**Fig. 5:**
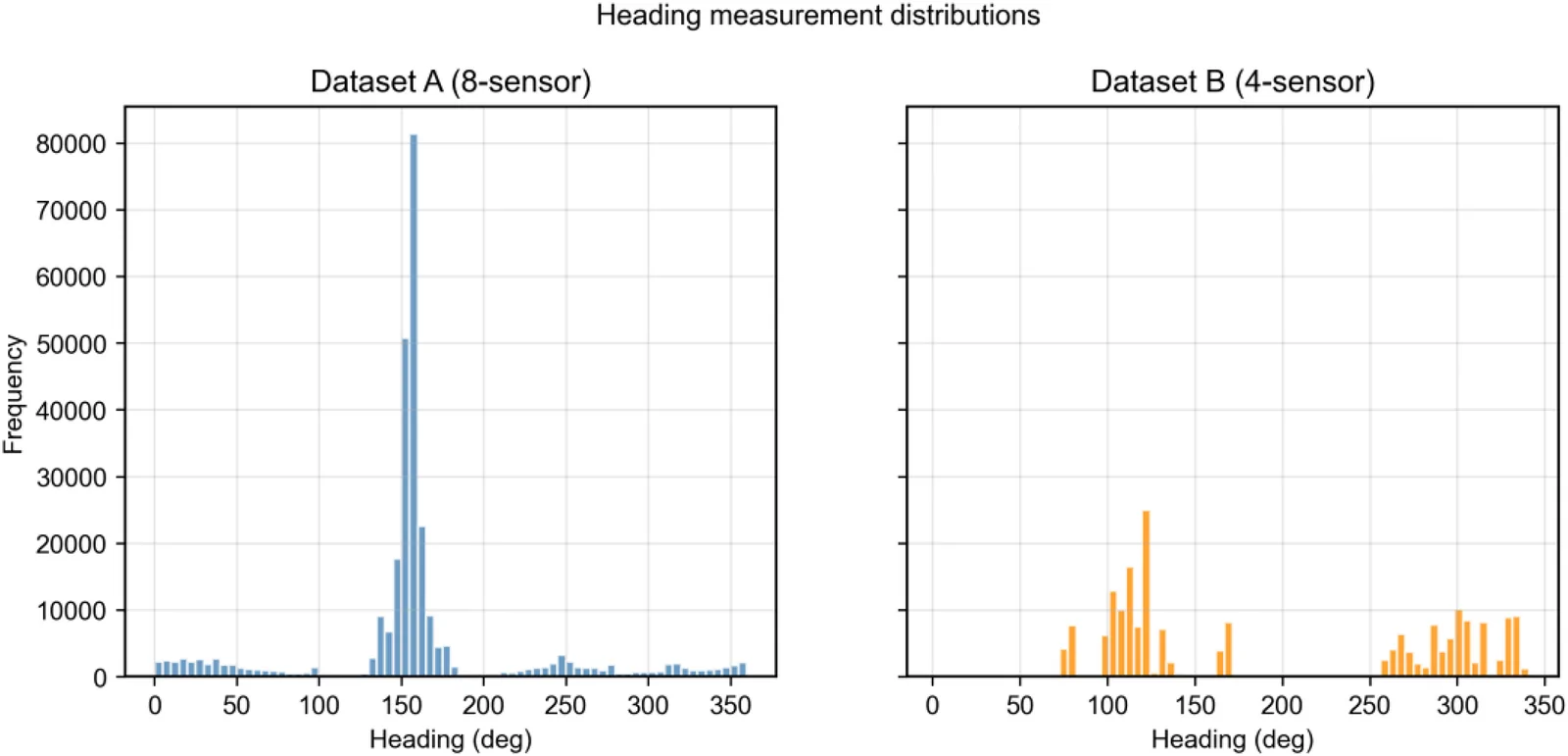
Heading measurement distributions for Dataset A (left) and Dataset B (right).

**Fig. 6:**
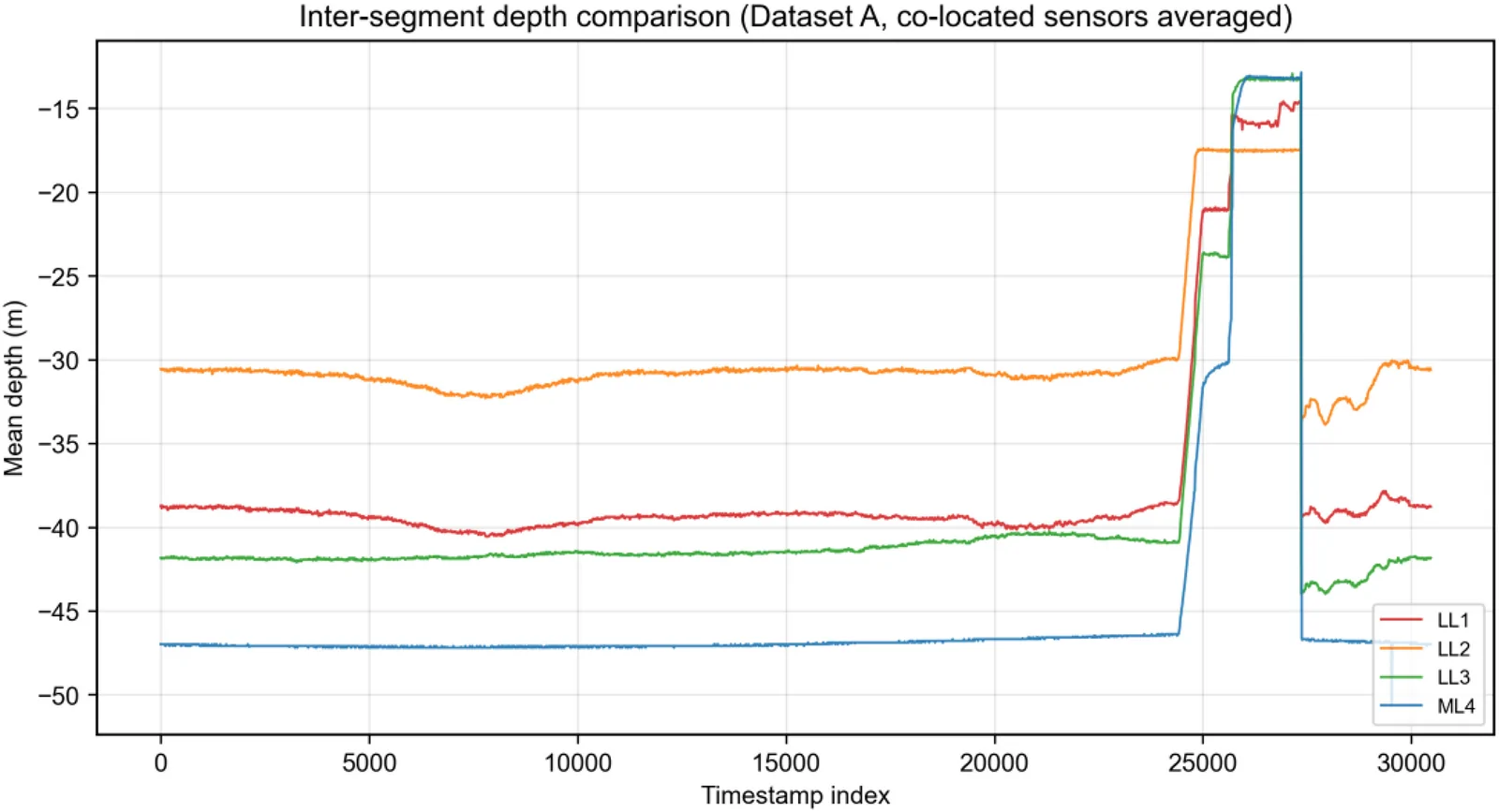
Inter-segment depth comparison for Dataset A (L1–L4), with co-located sensors averaged per timestamp.

To complement the tabular summaries in Table 9, Table 10, representative plots generated from files are provided in Fig. 3, Fig. 4, Fig. 5, Fig. 6. These visualizations illustrate depth trajectories, attitude distributions, and inter-segment comparisons, offering an immediate overview of deployment-scale behavior without specialized analysis software.

Depth time series for Dataset A are shown in Fig. 3, with each array segment (L1–L3: low-frequency; L4: mid-frequency) plotted separately. Segments L1–L3 operate at greater depths (approximately −13 m to −51 m), and temporal depth variations are visible across the ∼8.5 h recording interval.

Pitch distributions for both datasets are compared in Fig. 4. Dataset A exhibits a broader spread (SD ≈ 47.5°), consistent with greater array motion during the deeper multi-segment deployment, whereas Dataset B shows a narrower distribution (SD ≈ 2.6°) characteristic of the shallower mid-frequency configuration.

Heading distributions are presented in Fig. 5. Both datasets span the full compass range (0°–360°), with Dataset A centered near ∼159° and Dataset B near ∼198°, reflecting orientation differences between deployment phases.

Fig. 6 compares mean depth trajectories across segments L1–L4 in Dataset A, with co-located sensor pairs averaged at each timestamp. The plot highlights inter-segment depth offsets and relative stability, supporting visual assessment of array geometry consistency over time.

### 4.7 Data quality validation

Prior to release, the following checks were performed on each CSV file:

• **Completeness check:** Verified sensor reporting frequency and consistency of channel counts across timestamps.
• **Range validation:** Screened depth, heading, pitch, and roll values against physically plausible ranges (depth: 0 to −60 m for screening; file-specific minima/maxima in Table 8; heading: 0°–360°; pitch: −90° to +90°; roll: −180° to +180°). Values outside nominal pitch limits were retained in the released files because they are present in the raw telemetry stream and may reflect sensor-specific conventions or transient measurement anomalies (e.g., Dataset B pitch minimum = −110.90°; one record).
• **Continuity check:** Assessed timestamp spacing against the nominal 1 s interval and identified intervals with missing or irregular sampling.

The outcomes of these checks are summarized in Table 11, applied to the released CSV files after removal of redundant identical records (duplicate rows sharing the same Reception Time and Sensor Name with identical measurements were deduplicated, retaining the first occurrence). Missing values were counted across Depth, Heading, Pitch, and Roll fields. Timestamp gaps > 1 s were counted on the sorted sequence of unique Reception Time values in each file.

**Table 11:** Data quality validation outcomes.

Metric	Dataset A	Dataset B
Missing values (measurement fields)	0	0
Duplicate (time, sensor) records	0	0
Timestamp gaps > 1 s (unique timestamps)	113	513
Invalid headings (outside 0°–360°)	0	0
Invalid depth (positive values)	0	0
Invalid pitch (outside ±90°)	0	1
Invalid roll (outside ±180°)	0	0

**Note:** Timestamp gaps > 1 s account for **0.37%** (113/30,479) of unique timestamps in Dataset A and **1.03%** (513/49,557) in Dataset B. These gaps are **distributed sporadically** throughout the records rather than concentrated in specific periods, and are attributed to the time-division multiplexing scheme used for telemetry transmission. They do not indicate sensor malfunction or data corruption. The single pitch value outside ±90° in Dataset B (−110.90°) is retained as a raw outlier.

### 4.8 Accompanying visualization tool

A browser-based visualization web application is provided as a supplementary utility to facilitate visual inspection and exploratory analysis of the CSV datasets without specialized commercial software. The source code is publicly available at https://github.com/tyrone1979/underwater (application directory: app/). The tool was developed using **React, TypeScript, and Three.js** (via React Three Fiber) and is built with **Vite**. It loads the released CSV files and provides:

**Simulation view:** an interactive 3D underwater scene showing sensor positions and orientations, with playback controls, adjustable speed, temporal seeking, and a synchronized telemetry panel (depth, heading, pitch, roll);

**Dashboard view:** an interactive statistical dashboard with charts and tables for depth trends, attitude summaries, correlations, and related exploratory plots.

The application runs locally in a modern web browser after standard Node.js setup (npm install and npm run dev in the app/ directory). No dedicated backend server or WebSocket streaming is required for CSV playback. Build instructions and usage notes are provided in the repository README.

**Example workflow for data loading**

The CSV files can be readily loaded and processed in standard data analysis environments. A typical workflow involves the following steps:

1. Read the CSV file using any standard data loading function (e.g., pandas.read_csv in Python, readtable in MATLAB, or read.csv in R);
2. Inspect the column structure using the definitions provided in Table 2;
3. Filter rows by Sensor Name to isolate data from a specific sensor of interest;
4. Generate time-series plots of depth, heading, pitch, or roll against Reception Time for visual inspection or further analysis.

Summary statistics can be computed by grouping data by Deployment Location or Sensor Name to examine inter-segment or inter-sensor variability. The documented column structure and raw data format facilitate straightforward integration into existing analysis pipelines without requiring specialized preprocessing.

### 4.9 Example data reuse and quality assessment

**Co-located sensor consistency.** Each deployment position carries two independent sensor units; comparing their synchronized records provides an internal check on telemetry reliability. Table 12 summarizes Pearson correlation coefficients and root-mean-square error (RMSE) for depth measurements between co-located pairs, aligned by Reception Time.

**Table 12:** Co-located sensor depth consistency (aligned by Reception Time).

Dataset	Segment	Co-located pair	Depth correlation	Depth RMSE (m)
Dataset A	L1	Y23_T23–Y24_T24	0.988	7.014
Dataset A	L2	Y25_T25–Y26_T26	0.993	0.481
Dataset A	L3	Y27_T27–Y28_T28	0.987	8.291
Dataset A	L4	Y29_T29–Y30_T30	0.998	7.030
Dataset B	L4	Y29_T29–Y30_T30	0.998	0.368
Dataset B	L6	Y31_T31–Y32_T32	0.996	0.902

Co-located depth measurements exhibit strong positive correlation (r = 0.987–0.998 across all pairs), with RMSE values below 1 m for Dataset B. Larger RMSE values in selected Dataset A segments (L1, L3, L4; 7.0–8.3 m) primarily reflect persistent depth offsets between paired sensors rather than disagreement in temporal variation patterns, as indicated by the high correlation coefficients. These results support internal consistency of the released telemetry records.

Moving-average smoothing example. To illustrate a simple reuse application, a 30-s centered moving-average filter (window w = 30 samples at ∼1 Hz) was applied to pitch measurements from sensor Y29_T29 in Dataset A, where $P_{t}=\left(1/w\right)\sum_{\left(i=t-w+1\right)}^{t}P_{i}$$P_{t}=\left(1/w\right)\sum_{\left(i=t-w+1\right)}^{t}P_{i}$.

**Fig. 7:**
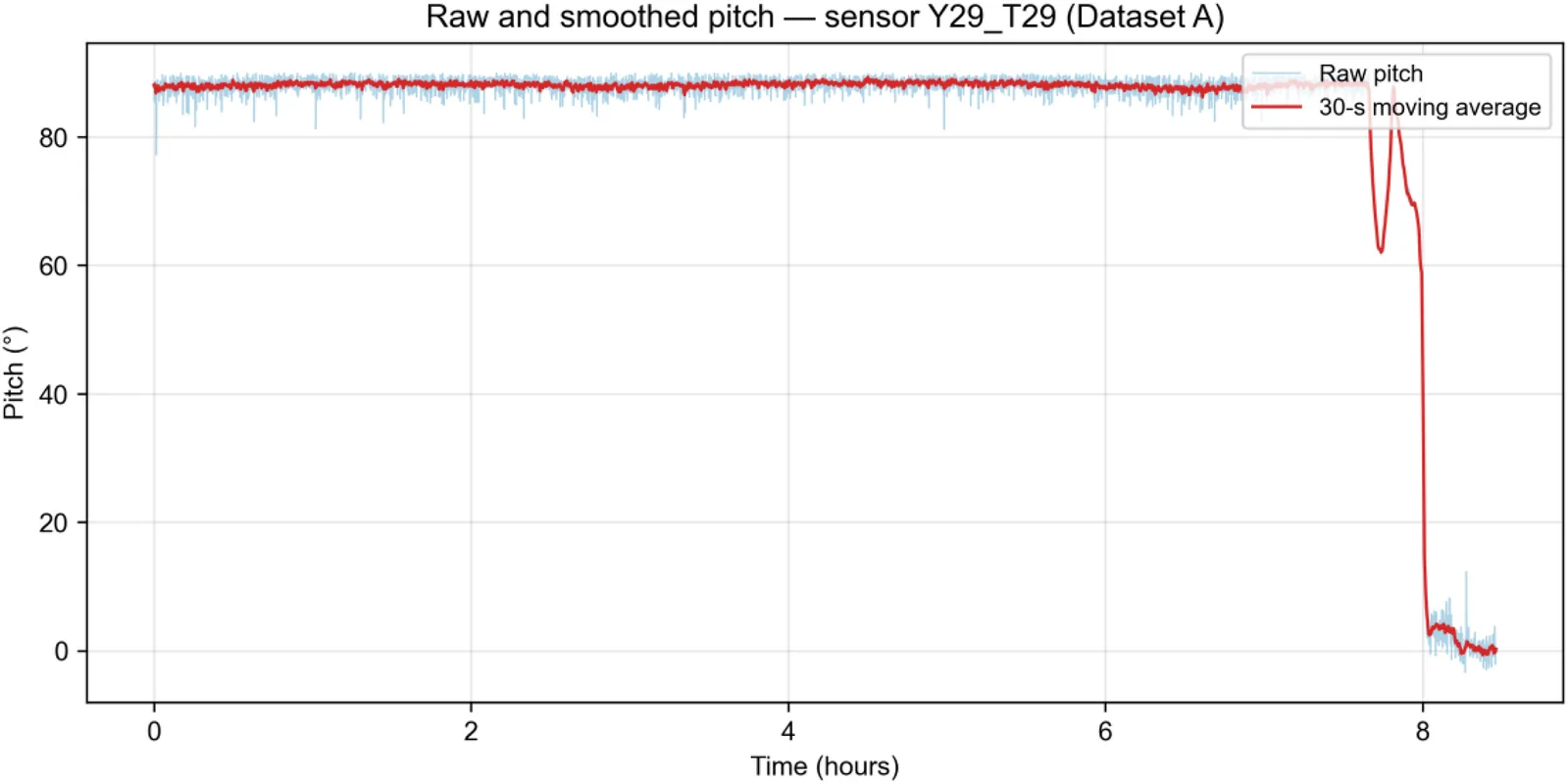
Raw and smoothed pitch measurements for sensor Y29_T29 (Dataset A). A 30-s centered moving-average filter (red) is overlaid on the raw 1 Hz pitch series (blue).

Fig. 7 compares the raw and smoothed pitch series in chronological acquisition order. Although the overall standard deviation remained similar (σ_raw = 19.68°; σ_smooth = 19.64°) because long-term orientation trends dominate the signal, the residual fluctuation level was reduced (residual SD = 1.13°; peak |raw − smoothed| = 11.35°), indicating suppression of short-term variations while preserving the deployment-mean pitch (82.73° before and after smoothing). Table 13 summarizes the smoothing metrics.

Together, the co-located consistency analysis and the moving-average example demonstrate that the dataset is suitable for telemetry quality assessment, attitude-smoothing benchmarks, and introductory sensor-fusion exercises without requiring specialized filtering algorithms.

## Limitations

The dataset has the following limitations:

**Spatial coordinates:** Absolute geographic coordinates (latitude and longitude) and precise relative X/Y/Z positions of individual sensors are not included. The CSV files provide deployment location identifiers (e.g., Low-frequency Array-L1–L3, Mid-frequency Array-L4, Mid-frequency Array-L6) but not exact along-array spacing between positions or offsets between co-located sensor pairs.

**Acoustic data:** The datasets contain only navigation and orientation telemetry (depth, heading, pitch, roll). Actual acoustic measurements such as sound pressure levels, frequency spectra, or acoustic arrival times are not included.

**Environmental metadata:** Water temperature, salinity, sound speed profiles, and sea state conditions during the deployment period are not provided.

**Sensor specifications**: Pre-deployment calibration coefficients, sensor manufacturer details, and instrument-specific accuracy metadata are not included.

**Deployment configuration:** The release comprises two related CSV files (Dataset B (underwater_sensor_array_4.csv) and Dataset A (underwater_sensor_array_8.csv)) representing different array configurations and water-depth conditions within a single continuous logging campaign. Results from one file should not be assumed to apply to the other without explicit comparison.

**Single campaign context:** The data represent one field logging campaign. Inter-deployment variability and long-term sensor drift cannot be assessed from these files alone.

**Timestamps:** Reception Time is stored as time-of-day only (no calendar date in the CSV). Calendar dates were omitted from the released files to satisfy operational confidentiality requirements associated with the field deployment. Consequently, the dataset cannot be correlated with external time-dependent environmental data, such as tidal cycles or diurnal patterns. This limits reconstruction of absolute timing across midnight or multi-day gaps without external deployment logs; relative timing within each file and the campaign wall-clock interval (08:08 Day 1 to 11:04 Day 2) are documented in Table 8.

## Ethics Statement

The authors have read and follow the ethical requirements for publication in Data in Brief. The current work does not involve human subjects, animal experiments, or any data collected from social media platforms.

## CRediT Author Statement

**Fujin Huang:** Conceptualization, Data curation, Investigation, Methodology, Software, Validation, Visualization, Writing – original draft; **Lei Zhao:** Writing – review & editing.
